# *HMOX1* Genetic Polymorphisms Display Ancestral Diversity and May Be Linked to Hypertensive Disorders in Pregnancy

**DOI:** 10.1007/s43032-022-01001-1

**Published:** 2022-06-13

**Authors:** Tianyanxin Sun, Giovanna I. Cruz, Nima Mousavi, Ivana Marić, Alina Brewer, Ronald J. Wong, Nima Aghaeepour, Nazish Sayed, Joseph C. Wu, David K. Stevenson, Stephanie A. Leonard, Melissa Gymrek, Virginia D. Winn

**Affiliations:** 1 Department of Obstetrics and Gynecology, Stanford University School of Medicine, Stanford, CA USA; 2 Department of Electrical and Computer Engineering, University of California San Diego, La Jolla, CA USA; 3 Department of Pediatrics, Stanford University School of Medicine, Stanford, CA USA; 4 Preeclampsia Foundation, Juneau Biosciences, LLC, Salt Lake City, UT USA; 5 Department of Anesthesiology, Perioperative and Pain Medicine, Stanford University School of Medicine, Stanford, CA USA; 6 Cardiovascular Institute, Stanford University School of Medicine, Stanford, CA USA; 7 Division of Vascular Surgery, Department of Surgery, Stanford University School of Medicine, Stanford, CA USA; 8 Department of Medicine, Department of Computer Science and Engineering, University of California San Diego, La Jolla, CA USA

**Keywords:** Hypertensive disorders in pregnancy (HDP), Ancestral diversity, *HMOX1*, Genetic variants, 1000 Genomes Project (1 KG), Preeclampsia (PE)

## Abstract

Racial disparity exists for hypertensive disorders in pregnancy (HDP), which leads to disparate morbidity and mortality worldwide. The enzyme heme oxygenase-1 (HO-1) is encoded by *HMOX1*, which has genetic polymorphisms in its regulatory region that impact its expression and activity and have been associated with various diseases. However, studies of these genetic variants in HDP have been limited. The objective of this study was to examine *HMOX1* as a potential genetic contributor of ancestral disparity seen in HDP. First, the 1000 Genomes Project (1 KG) phase 3 was utilized to compare the frequencies of alleles, genotypes, and estimated haplotypes of guanidine thymidine repeats (GTn; containing rs3074372) and A/T SNP (rs2071746) among females from five ancestral populations (Africa, the Americas, Europe, East Asia, and South Asia, *N* = 1271). Then, using genomic DNA from women with a history of HDP, we explored the possibility of *HMOX1* variants predisposing women to HDP (*N* = 178) compared with an equivalent ancestral group from 1 KG (*N* = 263). Both *HMOX1* variants were distributed differently across ancestries, with African women having a distinct distribution and an overall higher prevalence of the variants previously associated with lower HO-1 expression. The two *HMOX1* variants display linkage disequilibrium in all but the African group, and within EUR cohort, LL and AA individuals have a higher prevalence in HDP. *HMOX1* variants demonstrate ancestral differences that may contribute to racial disparity in HDP. Understanding maternal genetic contribution to HDP will help improve prediction and facilitate personalized approaches to care for HDP.

**Supplementary Information:**

The online version contains supplementary material available at 10.1007/s43032-022-01001-1.

## Introduction

Hypertensive disorders in pregnancy (HDP), including eclampsia (E), preeclampsia (PE), HELLP (hemolysis, elevated liver enzymes, low platelet count) syndrome, severe PE, chronic hypertension (CHTN), superimposed PE, and gestational hypertension (GHTN), impact about 8 million mother-infant pairs, and account for 14% of maternal mortality [1] and as high as 25% of perinatal mortality globally [2]. Racial disparity for overall maternal mortality has been reported with higher rates for Black women as well as for American Indian/Alaska Native women compared with other races [3, 4]. In addition, HDP contributes to the mortality rates in Black, American Indian/Alaskan Native, and Hispanic women compared with White women [4]. Similarly, a number of studies have shown that the prevalence of PE and related HDP is generally higher in Black women and lower in Asian or Pacific Islander women compared with White women and Hispanic women, but data are often limited for other races (reviewed in [5]). While studies are still limited in characterizing genetic factors or combinatory factors that predispose certain ancestral populations to HDP, it is well accepted that diverse genetic backgrounds [6] in addition to socioeconomic determinants [7] and racial inequities [5] likely play fundamental roles in contributing to the disparity observed in HDP prevalence, morbidity, and mortality.

Heme oxygenase 1 (HO-1, encoded by *HMOX-1*) is abundantly expressed in all nucleated cells and particularly in the liver, spleen, bone marrow, and placenta. HO-1 is highly expressed in the endothelium and has been suggested to impact endothelial dysfunction [8]. Endothelial dysfunction is recognized as an integral part of the spectrum of hypertensive diseases in pregnancy [9, 10]. HO-1 is induced in response to cellular insults such as inflammation and hypoxia. HO-1 catalyzes the degradation of heme to the anti-inflammatory and anti-apoptotic products of carbon monoxide (CO), and biliverdin, which in turn is reduced to the vasoactive and anti-oxidant product bilirubin [11]. Several genetic variants of *HMOX1* near its 5′ flanking region are known to be associated with a higher basal HO-1 expression as well as a more robust induced response. The shorter forms of the guanidine thymidine (GT) dinucleotide repeats (GT; containing rs3074372) and the A allele of A/T SNP (rs2071746) are considered more protective alleles, as these variants are associated with higher HO-1 expression and activity and associated with a lower risk of cardiovascular disease (reviewed in [11]). While the association of *HMOX1* variants with HDP is not as well characterized, the expression level of HO-1 has been explored in multiple studies. Lower HO-1 expression has been noted in the maternal blood [12] and placenta from PE pregnancies [13, 14] although there are conflicting findings from others, likely due to the heterogeneous nature of HDP. Furthermore, PE patients have been shown to have lower end-tidal breath CO levels [15, 16] consistent with having lower rates of heme degradation and HO-1 function. In a study in Finland, the LL (long long) GT genotype is associated with non-severe and late-onset PE [17]. Therefore, the genetically polymorphic *HMOX1* may serve as a promising genetic risk factor for HDP.

In this study, we first determined the allele frequencies, genotypes, and estimated haplotypes of the *HMOX1* variants GT repeats (including rs3074372) and A/T SNP (rs2071746) in African (AFR), American (AMR), European (EUR), East Asian (EAS), and South Asian (SAS) ancestral populations using the 1000 Genomes Project (1 KG) database. This was followed by genotyping *HMOX1* of patients who had a history of HDP to explore the possibility of *HMOX1* variants predisposing to HDP by comparing EUR HDP women to the equivalent ancestral group from 1 KG.

## Materials and Methods

### Subjects

The 1000 Genomes Project (1 KG), an international research database that provides the most complete catalog of human genetic variations to date [18], was used to study ancestral diversity of *HMOX1* variants. Individuals who shared the same locations as their grandparents were sampled from five main populations: AFR, AMR, EAS, EUR, and SAS. Of the 2,504 individuals from phase 3 of the 1 KG, 1,271 females were included in this study. The Preeclampsia Registry is an online-based patient-facing registry operated by the Preeclampsia Foundation. Participants were enrolled between July 2013 and January 2016 and answered questionnaires until April 2020 regarding their pregnancies and gave permission to registry staff to collect their medical records for confirmation of diagnoses. The definition of PE includes new-onset hypertension (systolic blood pressure ≥ 140 mmHg or diastolic pressure ≥ 90 mmHg) on two separate occasions after 20 weeks of gestation. Though proteinuria is no longer a requirement for current diagnosis in the presence of other end-organ damage (including thrombocytopenia, impaired liver function, new development of renal insufficiency, pulmonary edema, or cerebral/visual disturbances) [19], proteinuria was still used as a hallmark of PE in this study given the time frame of data collection, keeping diagnoses consistent within the cohort. Eclampsia is characterized by PE symptoms plus one or more seizures during or shortly after pregnancy. HELLP syndrome is defined by presentations of all 3 features (hemolysis, elevated liver enzymes, and low platelet count). Severe PE refers to PE with severe features including systolic blood pressure ≥ 160 mmHg or diastolic pressure ≥ 100 mmHg, or any one or more presentation(s) of HELLP syndromes. GHTN is BP elevation after 20 weeks of gestation in the absence of proteinuria or new-onset systemic syndromes listed for PE. CHTN is hypertension that predates pregnancy. Superimposed PE is CHTN in association with PE. Patients were assigned to the category of their most severe form of HDP if they were multipara. Participants also have provided a saliva or cheek swab sample for genomic DNA extraction for genetic studies. Of the 204 samples provided by the registry, 202 had sufficient DNA quality and quantity. Of these, 178 patients with confirmed EUR ancestry were included in the current study. The Preeclampsia Registry protocol is approved by Advarra institutional review board (protocol number Pro00008369).

### Ancestry Assignment

Participants in the 1 KG database reside in the same geographic locations as their grandparents. Their self-reported race information has been largely confirmed by ancestry classification analysis using SNP genotyping data, through KMeans clustering, PCA, or a combination of both methods [20]. EUR, EAS, and SAS groups were classified with a 100% precision rate and the AFR group was classified with 86.44–99.35% precision rates, whereas the AMR group was poorly classified (< 40% precision rate), with an overlap with either EUR or SAS group depending on clustering methods. For women with a history of HDP from the Preeclampsia Registry, self-reported race/ethnicity information was recorded. In addition, common markers (minor allele frequency [MAF] > 0.05) from the whole exome sequencing were used which resulted in ~ 30,000 SNPs and the genotype data were applied for PCA analysis to account for population stratification. EIGENSTRAT [21] was used to infer the axes of variation and the top 10 eigenvectors were analyzed, with most variance observed in the first and second eigenvectors/components. Using this method, a total of 178 women were classified as having EUR ancestry (171 of whom were self-reported White), while the rest did not have whole exome sequencing or subsequent PCA analysis data available.

### *HMOX1 Genotyping (*for *1 KG)*

DNA genotyping for *HMOX1* variants on subjects from 1 KG was carried out as follows: the entire GT repeats region encompasses rs3074372 (a GT repeats microsatellite), which is separated by an A/G SNP rs13057211 from another GT repeats region upstream (coordinates: 35776827–35776887 [hg19]; http://webstr.ucsd.edu). This entire region, not just rs3074372, is referred here as the GTn variant. The GTn genotypes of female samples from each main ancestral population of 1 KG were determined using haplotype inference and phasing for short tandem repeats (HipSTR v0.6.2) [22]. Comma separated list of cram files for female samples of each population was supplied using --bam flag (supports cram files as well) of HipSTR. Regions file for HipSTR only included Human_STR_900863 repeat (*HMOX1* GT repeats) from the HipSTR variant catalog: https://github.com/HipSTR-Tool/HipSTR-references/raw/master/human/hg38.hipstr_reference.bed.gz. Reference genome build hg38 was used in variant calling using HipSTR. Calls were filtered using the HipSTR filtering script https://github.com/tfwillems/HipSTR/blob/master/scripts/filter_vcf.py with suggested options (min-call-qual 0.9, max-call-flank-indel 0.15, max-call-stutter 0.15, min-call-allele-bias -2, min-call-strand-bias -2 according to https://github.com/tfwillems/HipSTR#call-filtering). A/T SNP (rs2071746) allele frequencies and genotypes of female individuals from 1 KG were directly downloaded from http://useast.ensembl.org/Homo_sapiens.

### *HMOX1 Genotyping (*for *HDP cohort)*

HMOX1 genotyping of genomic DNA from patients in PE registry (HDP cohort) was carried out as follows: GTn genotypes were determined using PCR and fragment analyses, modified from a previous study [23]. Briefly, the GTn region was amplified by PCR using primer pairs with 5′ FAM (fluorescein)-labeled forward primer (F: 5′-6-FAM-AGAGCCTGCAGCTTCTCAGA-3′, R: 5′-ACAAAGTCTGGCCATAGGAC-3′), and the size of the labeled PCR product was determined by fragment analyses using ABI 3130 × Genetic Analyzer and Peakscanner (Thermo Fisher Cloud), with GeneScan-500ROX (Applied Biosystems) as a size marker. The length of GTn for each allele was determined using formula *n*(GT repeat) = (*X*-67)/2–1, *X* being the length of the PCR product. The length of GTn was defined as short (*S*, *n* ≤ 27) or long (*L*, *n* > 27) based on its distribution in the study population. About 20% of the samples were randomly selected to run two independent PCR reactions, which confirmed accuracy and reproducible sizing. A/T SNP (rs2071746) of patient samples was genotyped using TaqMan SNP genotyping assay (Cat 4351379, Applied Biosystems), 7900HT Real-Time PCR system, and TaqMan Genotyper software following the manufacturer’s instructions. Haploview 4.1 [24] was used to perform haplotype analyses, including accessing linkage disequilibrium and haplotype-HDP association analysis, with GTn variant being simplified as a SNP variant [23].

### Statistical Analyses

Pearson Chi-square test was used for analyses of GTn alleles (short/long), A/T SNP alleles, and their corresponding genotypes between five populations from 1 KG followed by post hoc pairwise comparisons between five populations with Bonferroni adjustment (*p* < 0.005 was considered statistically significant). Fisher’s exact test was used for binary genotype analyses (e.g., AA vs. non-AA), also with Bonferroni adjustment (*p* < 0.005). To compare allelic frequencies and genotypes between EUR from 1 KG and the EUR HDP cohort, Fisher’s exact test was applied for all allele and genotype comparisons given the moderate sample sizes (*p* < 0.05 was considered statistically significant), and odds ratios were calculated. Stata 17 was used for all the above analyses. Haploview 4.1 [24] was used to perform haplotype analyses, including accessing linkage disequilibrium (LD) and haplotype-HDP association analysis, with GT variants being simplified as SNP variants [23]. Fisher’s exact test was also applied on the analyses of combinatory genotypes and haplotypes between 1 KG EUR and the HDP cohorts, using elsewhere as the reference group for analyses and calculation of odds ratios using SAS 9.4. The *p* threshold was corrected with Bonferroni adjustment (*p* < 0.0056 was used for nine comparisons of combinatory genotypes, *p* < 0.0125 was used for four haplotype comparisons). Minor allele frequency (MAF) and heterozygosity were computed using Haploview, and deviation from Hardy–Weinberg equilibrium (HWE) was tested (*p* < 0.001 indicates disequilibrium).

## Results

**Fig. 1:**
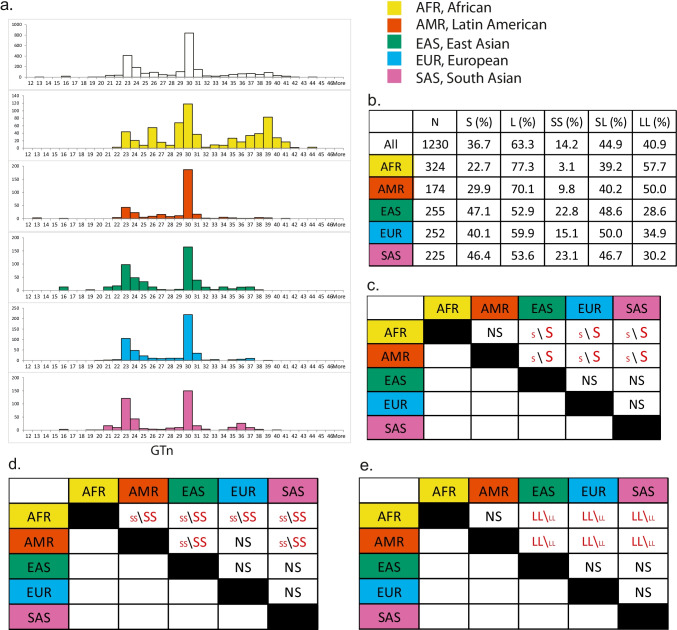
Ancestral diversity and the distribution of *HMOX1* GT repeats. **a** Histograms showing distribution of *HMOX1* GT repeats from each and all of the 1 KG populations. **b** Allele, genotype frequencies of GTn alleles, and the number of individuals from each population. **c** Pairwise comparisons of short GT (S) allele between populations using Pearson Chi-square test. **d** Pairwise comparisons of the SS genotype (vs. non-SS or L carriers) between populations using Fisher’s exact test following significant Chi-square test result among five populations. **e** Pairwise comparisons of the LL genotype (vs. non-LL or S carriers) between populations using Fisher’s exact test following significant Chi-square test result among 5 populations. For all pairwise comparisons, a Bonferroni correction was applied and *p* < 0.005 was considered statistically significant. *N*, number of subjects. *S*, short GT allele, *n* ≤ 27. *L*, long GT allele, *n* > 27. Each fill color was designated to a population. Red text denotes significant differences, and the font size indicates directionality of differed frequencies between any two given groups. Figure was made using Adobe Illustrator CS6, with colors that are unambiguous to color-blinds (https://jfly.uni-koeln.de/color/)

**Fig. 2:**
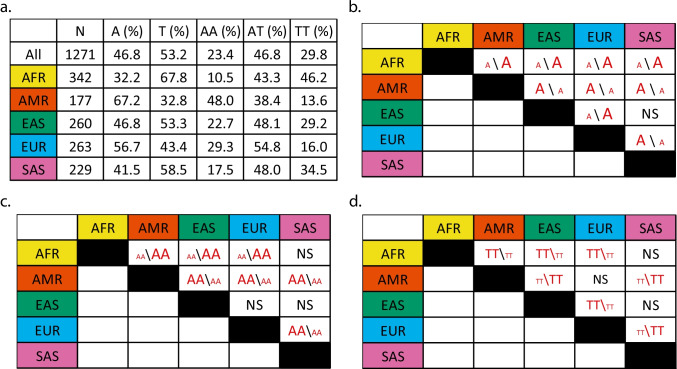
Ancestral diversity in A/T SNP of *HMOX1*. **a** Allelic and genotype frequencies of A variant and the number of individuals from each and all of the populations. **b** Pairwise comparisons of the A allele between populations using Pearson Chi-square test. **c** Pairwise comparisons of the AA genotype (vs. non-AA or T carriers) between populations using Fisher’s exact test. **d** Pairwise comparisons of the TT genotype (vs. non-TT or A carriers) between populations using Fisher’s exact test. For all pairwise comparisons, a Bonferroni correction was applied and *p* < 0.005 was considered statistically significant. *N*, number of subjects. Each fill color was designated to a population. Red text denotes significant differences, and the font size indicates directionality of differed frequencies between any two given groups. Figure was made using Adobe Illustrator CS6, with colors that are unambiguous to color-blinds (https://jfly.uni-koeln.de/color/)

Both *HMOX1* variants showed ancestral diversity among the five populations from 1 KG (Figs. 1, 2). The GTn was distributed in a bimodal pattern (with peaks at *n* = 23 and 30) in all populations except for AFR, which had a more widespread distribution pattern, especially with an additional peak around *n* = 39 (Fig. 1a). A total of 1230 of 1271 individuals passed the filters of GT sequences with informative calls (Fig. 1b). As shown in Fig. 1c, the S allele (linked to higher HO-1 expression than the L allele) was more common in EAS, SAS, and EUR women and the L allele was more common in AFR and AMR women, while no statistical differences were observed between AFR and AMR and among EAS, SAS, and EUR. Comparisons on binary genotypes—SS versus L carriers (LL + SL), or LL versus S carriers (SL + SS)—revealed that AFR had the lowest SS frequency of all groups (Fig. 1d) and Asian women (EAS and SAS) had higher frequencies of SS compared to AMR, while no statistical differences were observed among EAS, SAS, and EUR and between AMR and EUR. As a presumably high-risk genotype linked with lower HO-1 expression, LL was shown to be more common in AFR and AMR women (Fig. 1e).

Similarly, ancestral diversity was also observed for A/T SNP within the 1 KG, with the allelic and genotype frequencies summarized in Fig. 2a. The A allele (linked to higher HO-1 expression than the T allele) was the highest in AMR while it was the lowest in AFR. EUR had a higher frequency of the A allele compared with Asian women either EAS or SAS (Fig. 2b). AMR had the highest frequency of AA, while AFR women had a lower frequency of AA than every other group except for the SAS group (Fig. 2c). For the TT genotype (linked to lower HO-1 expression), AFR women had a higher frequency than AMR, EAS, and EUR. The two Asian groups EAS and SAS had higher frequencies of TT compared with either AMR or EUR (Fig. 2d).

**Fig. 3:**
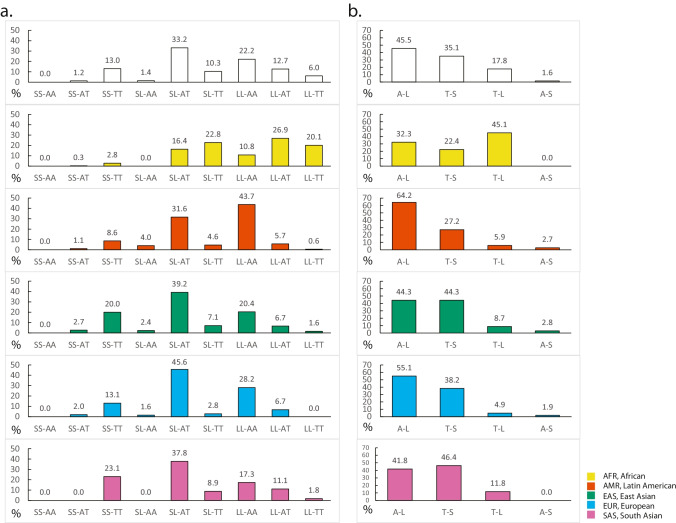
Combinatory genotypes and co-segregation of *HMOX1* variants among ancestries. **a** Histograms showing frequencies of nine combinatory genotypes of *HMOX1* among five populations as well as all combined. **b** Frequencies of estimated haplotypes (four types) among populations. Each fill color was designated to a population. *S*, short GT allele, *n* ≤ 27. *L*, long GT allele, *n* > 27. Figure was made using Adobe Illustrator CS6

Observations on the nine combinatory genotypes of both *HMOX1* variants showed that the most common genotype in all populations combined was SL-AT, while the least common genotype was SS-AA, which was completely absent from any 1 KG population examined (Fig. 3a, Supplementary Fig. 1). In addition, SS-AT or SL-AA, which is homozygous for either S or A variant, was rare across the board. For AMR, EAS, EUR, and SAS, several genotypes including SL-AT, LL-AA, and SS-TT appeared to be dominating across ancestries (Fig. 3a). However, genotypes in AFR seemed more widespread, showing high frequencies of LL-TT, LL-AT, and SL-TT while their counterparts appeared much rarer in the other populations (Fig. 3a). Further analyses of the genotypes were limited due to sample size within each combinatory genotype of any given population. However, given the proximity of these two variants, we also accessed the haplotypes (Fig. 3b) and potential ancestral differences in allelic association or LD (Supplementary Fig. 2a). A-L and T-S were the two most dominant haplotypes in AMR, EAS, EUR, and SAS, whereas T-L and A-L were most dominant in AFR. Strong evidence of LD was shown in all groups (*D*’ ≥ 0.9, *r*^2^ ≥ 0.6) except for AFR (*r*^2^ = 0.1, Supplementary Fig. 2a). All populations examined were in Hardy–Weinberg equilibrium (HWE) for both variants studied (*p* > 0.001), with T and L being the major alleles of the two variants, respectively (Supplementary Fig. 3).

Given that Black women have an elevated risk of HDP and AFR ancestry has high frequencies of the LL and TT genotypes, we next determined, utilizing genomic DNA provided by the Preeclampsia Foundation, if these genotypes were also higher in women who developed HDP. The registry individuals were predominantly of EUR ancestry; therefore, we limited the analyses to the EUR HDP patients exclusively (*N* = 178) and the ancestrally comparable reference group of 1 KG project (EUR, *N* = 263). Basic demographics and clinical characteristics of women with HDP using the most severe form of HDP experienced (if they had more than one pregnancy) are summarized in Table 1. Of note, HELLP and severe PE were over-represented in the registry population compared to anticipated prevalence of the different HDP classifications seen in the general population.

**Table 1:** Demographic and clinical characteristics of the EUR participants in the HDP cohort

Characteristic	Value	*N*^a^
Maternal age at delivery (years), mean ± SD	29 ± 5	164
Gravidity, median (min, max)	1 (1, 6)	164
Parity, median (min, max)	0 (0, 3)	164
Maternal ancestry, EUR (%)	100%	178
Pre-pregnancy BMI, mean ± SD	25.5 ± 5.1	172
GA at delivery (days), mean ± SD	237 ± 32	177
Delivery mode	Vaginal 30.5%, cesarean 69.5%	177
Number of babies	Singleton 94.4%, twins 5.6%	178
Fetal sex	Female 46.3%, male 53.7%	177
Baby length (In), mean ± SD	17.1 ± 3.0	158
Birth weight (oz), mean ± SD	72.1 ± 33.9	175
Eclampsia (*n*, %)	4, 2.3%	176
HELLP (*n*, %)	72, 40.9%	176
Severe PE (*n*, %)	79, 44.9%	176
Superimposed PE (*n*, %)	4, 2.3%	176
PE (*n*, %)	15, 8.5%	176
CHTN (*n*, %)	0, 0%	176
Gestational HTN (*n*, %)	2, 1.1%	176

^a^Numbers of total available values for each variable. Due to incomplete medical records retrieved for two patients to determine HDP classifications, a total of *N* = 176 were used to calculate percentages, but both patients had HDP on record

**Fig. 4:**
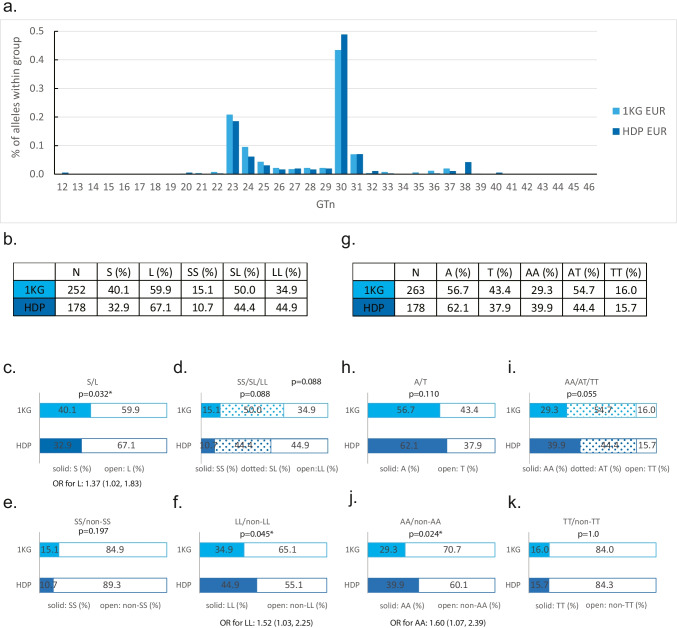
*HMOX1* variants are distributed differently in HDP patients. **a** Histogram showing distribution of *HMOX1* GTn from EUR population of 1 KG (reference) and a EUR HDP cohort. **b** Group sizes, allelic and genotype frequencies of GTn within the HDP cohort and reference group. **c**, **d** Stacked bar graphs comparing between the HDP cohort and reference group on allelic (S/L) and genotype distributions (SS/SL/LL). **e**, **f** Stacked bar graphs comparing the HDP cohort with reference group on binary genotypes (SS vs. non-SS and LL vs. non-LL), respectively. **g** Group sizes, allelic and genotype frequencies of A/T variant within the HDP cohort and reference group. **h**, **i** Stacked bar graphs comparing the HDP cohort with reference group on allelic (A/T) and genotype distributions (AA/AT/TT), respectively. **j**, **k** Stacked bar graphs comparing the HDP cohort with reference group on binary genotypes (AA vs non-AA and TT vs. non-TT), respectively. *N*, patient number. *S*, short GT allele, *n* ≤ 27. *L*, long GT allele, *n* > 27. The *p* value is from Fisher’s exact test. OR, odds ratio, shown with 95% confidence interval. Sky blue, EUR women from 1 KG as reference, darker blue, EUR HDP cohort. Figure was made using Adobe Illustrator CS6

The comparisons between 1 KG EUR population and a EUR cohort with a history of HDP revealed a significantly higher frequency of the L allele (n > 27) in the latter (Fig. 4a, b, c) (OR: 1.37; 95% CI: 1.02, 1.83). Neither A/T allele nor the genotypes of either variant displayed statistically significant differences between the 2 groups (Fig. 4d, g, h, i). However, when we divided GTn genotypes into SS and SL + LL (L carriers) or LL and SL + SS (S carriers), at-risk genotype LL was significantly higher in HDP cohort compared to EUR comparison group (OR: 1.52; 95% CI: 1.03, 2.25), and S carriers were rarer in HDP cohort (Fig. 4e, f), supporting our hypothesis that the short GT allele may protect women against HDP. Interestingly, when we divided the A/T genotypes into AA, AT + TT (T carriers) or TT, AT + AA (A carriers), the AA genotype was actually more common in the HDP cohort compared with the control (OR: 1.60; 95% CI: 1.07, 2.39), and T carriers were less common in the HDP cohort (Fig. 4j, k), which would correspond with lower HO-1 expression.

**Fig. 5:**
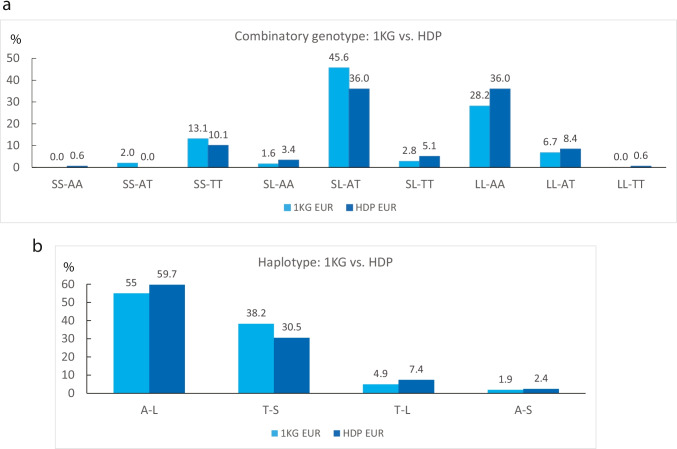
Combinatory genotypes of *HMOX1* variants in HDP patients and association between haplotypes and HDP. **a** Frequencies of nine combinatory genotypes of *HMOX1* in the EUR reference group (1 KG EUR) and the EUR HDP cohort. **b** Frequencies of estimated haplotypes (four types) within each group. Sky blue, EUR women from 1 KG as reference, darker blue, EUR HDP cohort. *S*, short GT allele, *n* ≤ 27. *L*, long GT allele, *n* > 27

For the combinatory genotypes of *HMOX1* in the EUR HDP cohort, the most common genotype was SL-AT, consistent with that in 1 KG EUR. SS-AA, as one of the least common genotypes, however, was not completely absent in the HDP cohort (Fig. 5a, Supplementary Fig. 1b, *N* = 1, *n* of GT = 19, 26). Similar to 1 KG EUR cohort, the HDP cohort exhibited low frequencies of the LL-TT (0.6%), SS-AT (0%), and SL-AA (3.4%) (Fig. 5a). Haplotype distribution of the HDP cohort mostly resembled that of the 1 KG EUR as well (Fig. 5b). Comparisons between the case group (HDP EUR) and control group (1 KG EUR) demonstrated no statistically significant differences for any of the combinatory genotypes (*p* > 0.0056, Fig. 5a, Supplementary Fig. 1b) or haplotypes (*p* > 0.0125, Fig. 5b, Supplementary Fig. 2b), with respective *p* thresholds adjusted for multiple comparisons. However, the SL-AT genotype (*p* = 0.048) and T-S haplotype (*p* = 0.025) suggested a trend of being negatively associated with HDP. As expected for the 2 EUR groups, the two *HMOX1* variants were in LD (*D*’ = 0.9, *r*^2^ = 0.7, Supplementary Fig. 2b).

## Discussion

Racial disparities clearly exist for the morbidity and mortality of HDP, with Black women and American Indian/Alaska Native women having a higher risk, while Asian women have a lower risk compared with White women [3, 4, 5], though minority groups were historically underrepresented. This study demonstrated the ancestral differences in *HMOX1* genetic polymorphism may potentially account for racial disparity in risk seen in HDP. AFR women have a higher frequency of the *HMOX1* variants associated with lower levels of HO-1 expression among the ancestry groups examined. For the GTn, AFR has the highest representation of the L allele, while Asian and EUR women have higher frequencies of the S allele (corresponding to higher HO-1 expression) as well as the S carriers than others, which is consistent with the clinical observations that Black women have elevated risk of HDP and Asian and White women have an overall decreased risk of HDP. For the A/T variant, AFR women had a higher frequency of the TT (linked to lower HO-1 expression) than most others, thereby potentially contributing to their risk for HDP. Interestingly, the A allele and the AA genotype (linked to higher HO-1 expression) are both the most abundant in AMR women, suggesting that AMR may be protected to some extent by the enrichment of this variant. This is supported by epidemiological studies showing comparable levels of HDP risks between AMR and EUR women, both lower than that of AFR women [25, 26]. However, the findings on AMR women need to be interpreted with caution given the heterogeneous nature of AMR group, as this group from 1 KG was the least well classified with < 40% precision using SNP genotyping data [20]. Interestingly, the T allele and TT genotype (linked to lower HO-1 expression) are more common in Asian women (both EAS and SAS) compared to EUR. Given the overall lower risk of HDP in Asian women than White women, it is plausible that the lack of protection resulting from high frequency of the T allele may be compensated by the protection from their enriched S allele. Notably, haplotype analyses revealed a strong allelic association between these two variants, with A-L and T-S being the most common haplotypes in all ancestral groups except for AFR, which could partially explain why both the T and S alleles are enriched in Asian populations. It is also worth noting that the AFR women had a greater variety of GTn lengths and abundant combinatory genotypes, which might indicate a greater genetic diversity in the AFR population at the levels of microsatellites as well as genotypes. This is not surprising given the population structure and the substantial genetic diversity in the AFR populations stemming from their deep evolutionary history [27], and populations with AFR ancestry are known to harbor the greatest numbers of variant sites [18]. Whether or not this expanded diversity in the AFR women is associated with a greater spectrum of disease presentations in HDP requires further studies. In addition, the haplotype distribution pattern is also strikingly different with T-L (both T and L are linked to lower HO-1 expression) as the most frequent haplotype, and yet only a weak allelic association was found in the AFR women (*r*^2^ = 0.1), unlike women of any other groups. This is in line with findings that the greater genetic diversity in AFR populations leads to a larger number of haplotypes and that the decay of LD as a function of physical distance is the fastest in AFR populations [18].

Given the disparity in HDP and the diversity in *HMOX1* genetic polymorphisms among ancestral groups, we then determined if the genetic differences were also reflected in women who developed HDP. By comparing a EUR HDP cohort with a reference group (1 KG EUR), we found that a short GT expansion likely is protective against HDP as the L allele and the LL genotype are significantly enriched in the HDP cohort. However, for the A/T variant, no differences were observed at the allelic level in this EUR HDP cohort, consistent with the finding from a recent study on the A/T SNP (rs2071746) of *HMOX*, also revealing no significant differences in its polymorphisms between PE and control groups in a Chinese population [28]. However, the AA genotype is more enriched in our HDP cohort compared with the ancestral reference group. This could again be due to the LD of these two *HMOX1* variants, given that EUR is one of the ancestral groups that harbor the A-L and T-S as the most common haplotypes. From analyses on each variant separately and in view of their allelic association, the GTn variant seems more likely to contribute to the risk of HDP than the A/T variant*.* Regarding combinatory genotypes and haplotypes, though the differences in HDP cohort did not reach statistical significance after adjustment for multiple comparisons, it is still of interest that the T-S haplotype (*p* = 0.025) or the SL-AT genotype (*p* = 0.048) displayed a tendency to be lower in HDP patients. Certainly, a larger cohort and a more in-depth haplotype analysis model considering various GTn lengths are necessary to better understand the role of *HMOX1* variants in predicting HDP for individuals. Also, analysis done for the other ancestral groups, particularly the AFR, could be more informative.

There are several limitations to this study. First, patient recruitment through the Preeclampsia Registry was biased towards patients who had severe forms of HDP and unfortunately did not have an adequate size control group (no history of HDP). Therefore, for a comparison group, we utilized the 1 KG to serve as a reference group of EUR from the general population. However, the 1 KG lacks available clinical pregnancy outcomes so likely includes women with HDP, which could be 3–5% given a baseline risk of HDP. In terms of our results, this would only dilute the ability to detect any difference. Since the HDP cohort is skewed to the most severe HDP subtypes, the potential contribution of *HMOX1* variants reflects these more severe forms of HDP and contribution of risk to more mild forms of HDP cannot be determined. Secondly, the HDP cohort had limited representation of ancestral groups other than European (23 of 201 women were not European); therefore, we were not able to analyze contributions of *HMOX1* variants to HDP risk directly for these other ancestry groups. A diverse prospectively collected pregnancy cohort with known obstetrical clinical outcomes could better elucidate *HMOX1* contribution to HDP risk in future studies.

Some studies reported lower HO-1 expression in the maternal and fetal compartments in PE [12, 13 14] while others reported conflicting findings [29, 30]. This is likely due to sample sizes, the heterogeneous nature of HDP, and other confounding factors. A partial deficiency of murine HO-1 leads to a condition that resembles PE with increases in diastolic pressures, fetal IUGR, and defects in the placental vasculature [31, 32]. Furthermore, the maternal genetic polymorphisms of the GT variants of *HMOX1* have been reported by others to be associated with non-severe PE and late-onset PE [17]. Kaartokallio et al. found that besides the maternal counterpart, fetal long GTn is also associated with PE but for early onset and severe PE subtypes [33]. This highlights the importance of considering both fetal and maternal *HMOX1* variants to delineate the roles of genetic factors in the maternal-placental-fetal physiology in future studies.

It has also been proposed that pregnancy can be metaphorically viewed as a car with an accelerator having exacerbated inflammation, oxidative stress, and an angiogenic milieu prone to imbalance, with the brake being endogenous protective pathways like HO-1/CO which, if malfunctional, eventually leads to adverse pregnancy outcomes, such as preeclampsia [34]. Maternal HO-1 plays a fundamentally protective role in endothelium health and endothelial dysfunction is a central feature of HDP despite the heterogeneity of its diverse clinical manifestations [9, 10], though other genes and their genetic polymorphisms involved in anti-oxidant defense capacity may also play important roles in maintaining endothelium health [35]. Factors coming from the placenta, such as sFlt1 (soluble fms-like tyrosine kinase-1) and sEng (soluble endoglin) believed to contribute to endothelial dysfunction, could be countered by the strength of the maternal endogenous protective mechanisms such as HO-1, ultimately impacting the degree of clinical manifestation. For example, a diseased placenta with strong maternal protection could result in no or mild disease, while a weak maternal protective system leads to more severe disease. It is important to recognize that HDP disease manifestations are multifactorial with contributions from both maternal and fetal/placental factors, as well as in the context of racial disparity and social determinants. Understanding ancestral genetic differences and how these may predispose women to HDP is clearly only one part of a larger picture.

## Conclusions

To our knowledge, this is the first study investigating the two *HMOX1* variants in the contexts of ancestral diversity among African, Latin American, European, East Asian, and South Asian populations and HDP. We report significant ancestral differences in *HMOX1* genetic polymorphisms among African, Latin American, European, East Asian, and South Asian women, highlighting that African ancestry appears to have a distinct distribution with greater diversity in *HMOX1* regulatory variants than other groups. The enriched *HMOX1* variants in the African population are associated with lower levels of HO-1 expression, which may contribute to a higher risk of HDP and other cardiovascular diseases. Linkage disequilibrium exists for the two *HMOX1* variants in all but African women; and of these two, GT variants were suggested to be the more relevant variant of *HMOX1* in considering HDP risk. Future studies inclusive of more groups, particularly underrepresented and admixed populations, and with a greater spectrum of HDP outcomes, are needed. Additionally, mechanistic studies focusing on the functionality of *HMOX1* variants in maternal endothelial systems would provide a more definitive answer to how *HMOX1* variants and HO-1 function impact HDP manifestation, especially given its responsiveness to therapeutic agents including aspirin [36] and statins [37], which can potentially help build a more personalized approach for both the prediction and treatment of HDP.

## Supplementary Information

Below is the link to the electronic supplementary material.

Supplementary file1 (EPS 1171 KB)

Supplementary file2 (EPS 1122 KB)

Supplementary file3 (EPS 1051 KB)

## Data Availability

The data that support the findings of this study are available upon request from the corresponding author.

## Code Availability

All code used for this study is publicly available and referenced in methods.

## Abbreviations

AFR: African
AMR: Latin American
BMI: Body mass index
CHTN: Chronic hypertension
CO: Carbon monoxide
E: Eclampsia
EUR: European
EAS: East Asian
GHTN: Gestational hypertension
GTn: Guanidine thymidine repeats
HDP: Hypertensive disorders in pregnancy
HELLP: Hemolysis, elevated liver enzymes, low platelet count
HO-1 (encoded by * HMOX1*): Heme oxygenase-1
HWE: Hardy-Weinberg equilibrium
HipSTR: Haplotype inference and phasing for short tandem repeats
IUGR: Intrauterine growth retardation
LD: Linkage disequilibrium
MAF: Minor allele frequency
PE: Preeclampsia
PCA: Principal component analysis
SNP: Single nucleotide polymorphism
SAS: South Asian
1KG: The 1000 Genomes Project
